# TRIM25 contributes to the malignancy of acute myeloid leukemia and is negatively regulated by microRNA-137

**DOI:** 10.1515/med-2021-0003

**Published:** 2020-12-24

**Authors:** Sheng Wang, Bang Shuo Zhang, Yi Yang, Ying Li, Jing Long Lv, Yu Cheng

**Affiliations:** Department of Hematology, Three Gorges Hospital Affiliated to Chongqing University, Chongqing, 404000, China; Department of Gastroenterology, Three Gorges Hospital Affiliated to Chongqing University, No. 165 Xincheng Road, Wanzhou District, Chongqing, 404000, China

**Keywords:** TRIM25, acute myeloid leukemia, miR-137, proliferation, metastasis

## Abstract

**Background:**

Acute myeloid leukemia (AML) is a ubiquitous malignancy that occurs in the hematological system. Tripartite motif-containing 25 (TRIM25) has been found to be involved in various carcinomas comprising AML. However, the function and underlying causative role of TRIM25 in AML are still obscure.

**Methods and materials:**

Quantitative real-time polymerase chain reaction (qPCR) was used for assaying TRIM25 and miR-137 expression in AML samples and cells. CCK-8 assay, Calcein-acetoxymethylester/propidium iodide staining, and Transwell assay were adopted to assay cell proliferation, invasion, and migration. Dual-luciferase reporter experiment was used for analyzing the interaction of TRIM25 with miR-137. Western blot was used for assaying protein expression levels.

**Results:**

This study confirmed that TRIM25 expression was upregulated in AML samples and cell lines, whereas miR-137 expression was downregulated. Overexpression of TRIM25 significantly contributed to AML cell’s proliferation, invasion, and migration, whereas knockdown exerted the opposite effect. In addition, TRIM25 was a downstream target of miR-137 in AML cells and negatively modulated by miR-137.

**Conclusion:**

TRIM25 was targeted and regulated by miR-137, exerted a carcinogenic function in AML, and could be used as a latent biomarker and a treatment target for AML.

## 1 Introduction

Acute myeloid leukemia (AML) is the most common hematological malignancy in the world blood system characterized by the proliferation and infiltration of clonal, abnormally differentiated hemopoietic stem cells in bone marrow, as well as peripheral blood, etc. [1]. AML is the most ubiquitous type of leukemia that occurs in adults, and its global incidence is about 162/1 million, which increases with age [2,3]. Although AML has made good progress in diagnosis, treatment, and prognosis, its overall survival is still less than 50% [4]. Thus, it is urgent to explore further the pathogenesis of AML to seek for novel therapeutic strategies.

Tripartite motif-containing 25 (TRIM25) is also called estrogen-responsive finger protein, and the encoded TRIM25 protein belongs to the tripartite motif (TRIM) family [5]. Many TRIM family proteins harbor the function of E3 ligase [6]. Researches evidenced that the TRIM family has antiviral effects and actively participates in modulating cell differentiation, proliferation, apoptosis, and tumorigenesis [7]. Studies have confirmed that the abnormal expression of TRIM25 is a crucial factor in the development of carcinoma. For example, TRIM25 knockdown not only restrains prostate carcinoma cell proliferation but also induces apoptosis via activating p53 protein [8]. The increased expression of TRIM25 in breast cancer is related to unfavorable overall survival [9]. Overexpression of TRIM25 contributes to lung carcinoma cell proliferation and migration [10]. Nonetheless, not only the role but also the potential mechanism of TRIM25 in AML is currently indistinct.

MicroRNA (miRNA) is a type of small RNA without coding function but has a length of 21–25 nucleotides. It is a significant factor in gene modulation by suppressing the translation and degradation of target RNA [11]. miRNAs have shown to modulate diverse biological signs of progress, including differentiation, apoptosis, and proliferation [12]. The research found that miRNA is a significant factor in distinguishing normal blood cells [13]. miRNA dysregulation can induce a variety of human diseases. The abnormal expression of miRNA is closely connected to cancer progression, making it a latent marker for carcinoma prognosis and diagnosis [14]. miR-137 belongs to the miRNA family and has been revealed to suppress cancer in various cancers’ progress. For example, miR-137 interferes with the interaction between BCL11A and DNMT1 to inhibit the stemness and tumorigenesis of triple-negative breast cancer cells [15]. miR-137 is inhibited by DNA hypermethylation and exerts a carcinoma inhibition effect in endometrial carcinoma [16]. Moreover, it was revealed that miR-137 is reduced in AML, and the increase of miR-137 suppresses AML cell growth and metastasis [17]. Notwithstanding, the specific causative role of miR-137 in the progress of AML has not been completely clarified, and therefore further research is still necessary.

This study explored the interaction between TRIM25 and miR-137 to reveal their potential mechanisms in the malignant progression of AML. The results of our research confirmed the upregulation of TRIM25 in AML. Moreover, TRIM25 expression is negatively modulated by miR-137, which participates in facilitating AML cell’s proliferation, invasion, and migration.

## 2 Materials and methods

### 2.1 Clinical samples and patients

A total of 45 patients with AML (20 males and 25 females, 50–72 years old, mean age 63.6 ± 5.1 years) and 45 healthy volunteers who donate bone marrow were regarded as normal controls (27 males and 18 females, 50–72 years old, mean age 63.8 ± 5.3 years) who were admitted at the Three Gorges Hospital of Chongqing University from January 2016 to June 2019. Investigation of the pathological and clinical follow-up data of the patients was completed before the operation. Before using these clinical samples, written informed consent was obtained from the research subjects. The experiments were approved by the Ethics Committee of the Three Gorges Hospital Affiliated to Chongqing University. All the patients provided written informed consent and the project was in accordance with the Helsinki Declaration of 1975.

### 2.2 Cell culture and transfection

AML cell lines (HEL, Kasumi-1, HL-60, MEG01 cells) and human bone marrow stromal cell line HS-5 were purchased from the Cell Bank of the Chinese Academy of Sciences (Shanghai, China) and were cultivated in DMEM (Invitrogen) comprising 10% FBS (Hyclone) and antibiotics (100 U/mL penicillin and 100 mg/mL streptomycin) (Hyclone) in a 5% CO_2_, 37°C, humidified incubator.

miR-137 mimic (miR-137), mimic negative control (miR-con), miR-137 inhibitor (miR-137-in), negative inhibitor control (miR-in), small interfering RNA TRIM25 (si-TRIM25#1, si-TRIM25#2, and si-TRIM25#3), negative control (si-NC), pcDNA3.0 (Vector), together with pcDNA-TRIM25 (TRIM25) were the products of GenePharma (Shanghai, China). The plasmids mentioned above or oligos were separately transfected into Kasumi-1 and HL-60 cells that cultivated to logarithm stage, applying Lipofectamine 2000 (Invitrogen, Shanghai, China). The transfected cells were available for the subsequent experiments.

### 2.3 Quantitative real-time polymerase chain reaction (qPCR)

According to (Invitrogen, Shanghai, China) instructions, the total RNA from AML cells or samples was extracted using TRIzol reagent. To detect TRIM25 mRNA expression, reverse transcription of the extracted RNA was performed to obtain cDNA by PrimeScript One-step RT-PCR kit (Takara). For miR-137 expression analysis, miScript reverse transcription kit (Qiagen) was adopted for synthesizing cDNA from 50 ng of extracted RNA. SYBR Green Master Mix (Takara) was applied for assaying gene expression levels. The relative expression was assessed by adopting the 2^−ΔΔCt^ method. β-Actin or U6 was recognized as a standardized internal control. The synthesis of PCR primers was completed (Sangon Biotech, China). The primer sequences are as follows: TRIM25 (forward 5′-GTCTCTACCCAGAACAGTTTCC-3′ and reverse 5′-ATCCAACACAGGCTGATTCC-3′); β-actin (forward 5′-ACTCGTCATACTCCTGCT-3′ and reverse 5′-GAAACTACCTTCAACTCC-3′); miR-137 (forward 5′-GCGCGCTTATTGCTTAAGAATAC-3′ and reverse 5′-GTCGTATCCAGTGCAGGGTCCGAGGTATTCGCACTGGATACGACCTACGC-3′); and U6 (forward 5′-CTCGCTTCGGCAGCACA-3′ and reverse 5′-AACGCTTCACGAATTTGCGT-3′). Three repetitions of all experiments were completed, in triplicate.

### 2.4 Cell proliferation assay

According to (Dojindo Molecular Technologies, Japan) instructions, cell proliferation was assayed using CCK-8. In a word, the injection of cells was realized in 96-well plates with 1 × 10^4^ cells per well and then cultivated in DMEM medium. At the end of the experiment, CCK-8 reagent (10 µL) was supplemented to the well. After incubating for 2 h under 37°C conditions, the absorbance measurement was accomplished at 450 nm using a microplate reader.

### 2.5 Fluorescence cell viability assay

Cell viability was detected by Calcein-acetoxymethylester/propidium iodide (Calcein-AM/PI) Double Stain Kit (American Type Culture Collection, VA, USA). Then 5 µL of Calcein-AM solution (2 mM) and 15 µL of PI solution (1.5 mM) were added to 5 mL of 1× Assay Buffer. As a result, the working solution concentration of Calcein-AM/PI was 2 and 4.5 µM respectively. The cells were later incubated for 15 min at 37°C. Photographs were taken using the fluorescence microscope.

### 2.6 Cell migration and invasion assay

Matrigel-coated Transwell and Transwell inserts (Corning, MA, USA) were applied for assaying the invasion and migration of AML cells. In brief, in 200 µL of serum-free medium, Kasumi-1 and HL-60 cells with a density of 2 × 10^4^ cells were added, either covered or not covered with Matrigel. Then, the cell plate was incubated under 5% CO_2_, 37°C, humidified environment. After cultivating for 24 h (migration test) or 48 h (invasion test), the upper chamber cells were mildly wiped off using a cotton swab. In the lower chamber, migrating or invading cells were fixed using alcohol for 15 min and stained using crystal violet for 10 min. Finally, the cells were counted using an optical microscope (Olympus, Tokyo, Japan) at 200×.

### 2.7 Western blot

According to the supplier’s instructions, RIPA lysis buffer (Beyotime) was used to isolate AML samples and cells’ total protein. The protein samples (20 µg) were separated using SDS-PAGE. Subsequently, the proteins were transferred to PVDF (Millipore, Billerica, MA). The membrane was blocked in 5% skim milk at room temperature for 2 h, and then incubated at 4°C with TRIM25 (Abcam) and β-actin (Abcam) overnight. After setting with HRP-linked secondary antibody at room temperature for 1 h, the protein bands were visualized using an electrochemiluminescence kit (Pierce Biotechnology), adopting β-actin protein as internal controls.

### 2.8 Dual-luciferase reporters assay

The 3′UTR sequence of TRIM25 was cloned into the pMIR eukaryotic expression vector (Promega) containing the luciferase gene for recovery, digestion, and purification to construct the pMIR-TRIM25-WT plasmid. Similarly, the pMIR-TRIM25-MUT plasmid was also built. Based on the manufacturer’s protocol, HL-60 cells were co-transfected with WT or MUT and miR-137 mimics or miR-con applying Lipofectamine 2000 reagent. Luciferase activity was assayed using the luciferase assay system (Promega) and was standardized to Renilla.

### 2.9 Statistical analysis

All the data were analyzed by GraphPad Prism 8.0 and SPSS statistical and presented as mean ± standard error (SD) from at least three individual experiments. Student’s *t*-test and one-way analysis of variance were applied to analyze the significance differences. Linear correlation between miR-137 and TRIM25 was calculated by Pearson’s correlation test. *P* < 0.05 was considered as statistically significant.

## 3 Results

### 3.1 The expression characteristics of TRIM25 in AML

**Figure 1:**
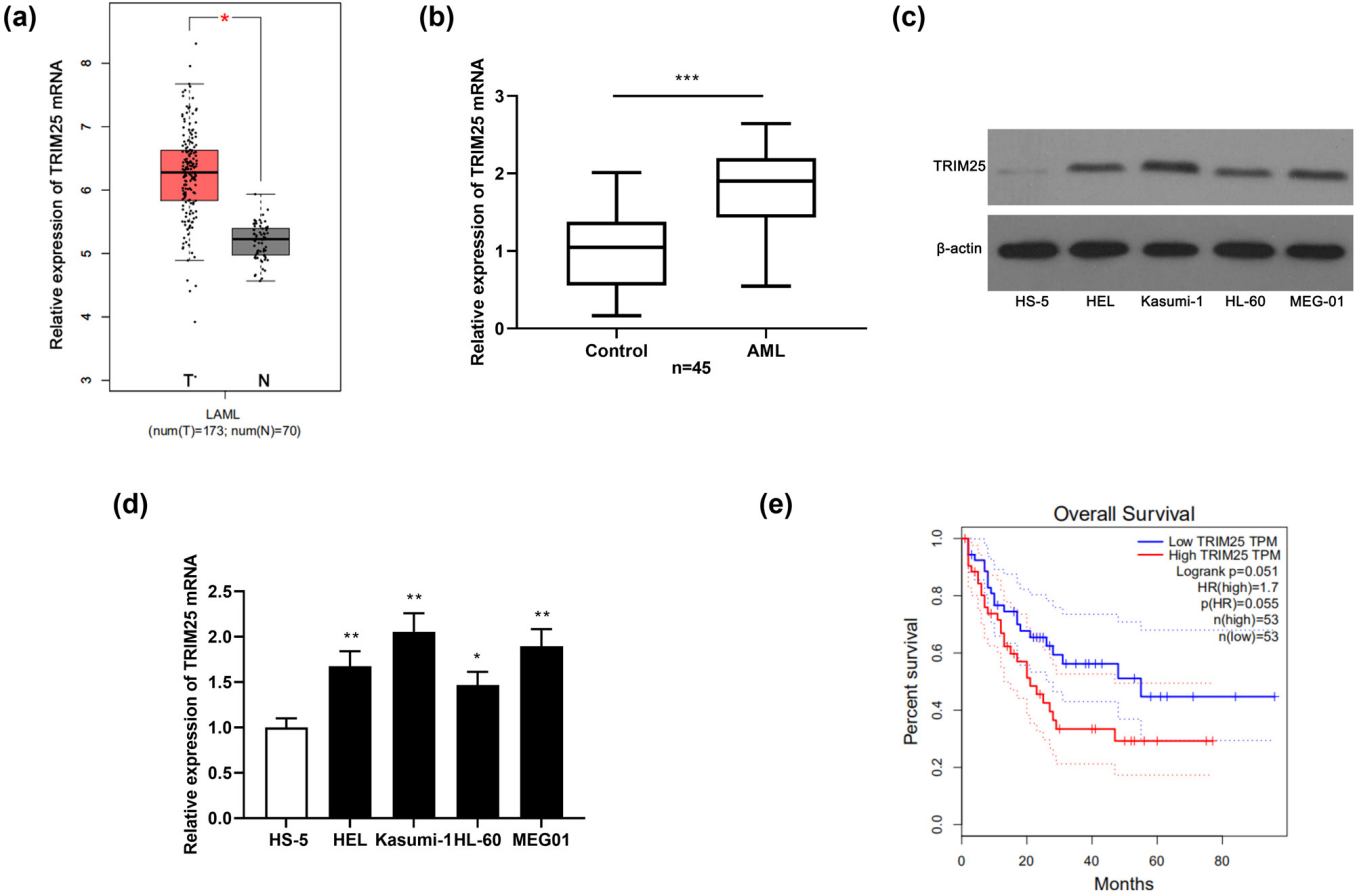
TRIM25 was highly expressed in AML samples and cells and was related to poor prognosis. (a) GEPIA database to retrieve TRIM25 expression in AML. (b) qPCR to assay TRIM25 mRNA expression in samples from 45 patients with AML and 45 healthy volunteers. (c and d) Western blot and qPCR to determine TRIM25 protein and mRNA expression in AML cells and HS-5 cells. (e) GEPIA database to analyze the correlation between the expression level of TRIM25 and the prognosis of patients with AML. *P < 0.05, **P < 0.01, ***P < 0.001.

To explore TRIM25 expression in AML, we first retrieved TRIM25 expression in the TCGA-AML sample through the GEPIA database (http://gepia.cancer-pku.cn/). As shown in Figure 1a, TRIM25 was significantly upregulated in the AML samples. Subsequently, qPCR was applied for assaying the TRIM25 mRNA level in the blood samples of 45 patients with AML and 45 healthy volunteers. It was discovered that the TRIM25 mRNA level in samples of patients with AML was strikingly upregulated in contrast to normal controls (Figure 1b). qPCR and western blot manifested that TRIM25 mRNA and protein expressions in AML cell lines were noteworthily higher than that of HS-5 cells (Figure 1c and d).

Moreover, we also searched the GEPIA database to understand the association of the high TRIM25 expression in AML with the patient’s prognosis. As a result, patients with AML with highly expressed TRIM25 harbored shorter overall survival than those with low expressed TRIM25 (Figure 1e). All the above results implicated that TRIM25 plays a carcinogenic role in AML and can be used as a potential marker of unfavorable prognosis in AML.

### 3.2 TRIM25 facilitated the AML cell’s proliferation, migration, and invasion

**Figure 2:**
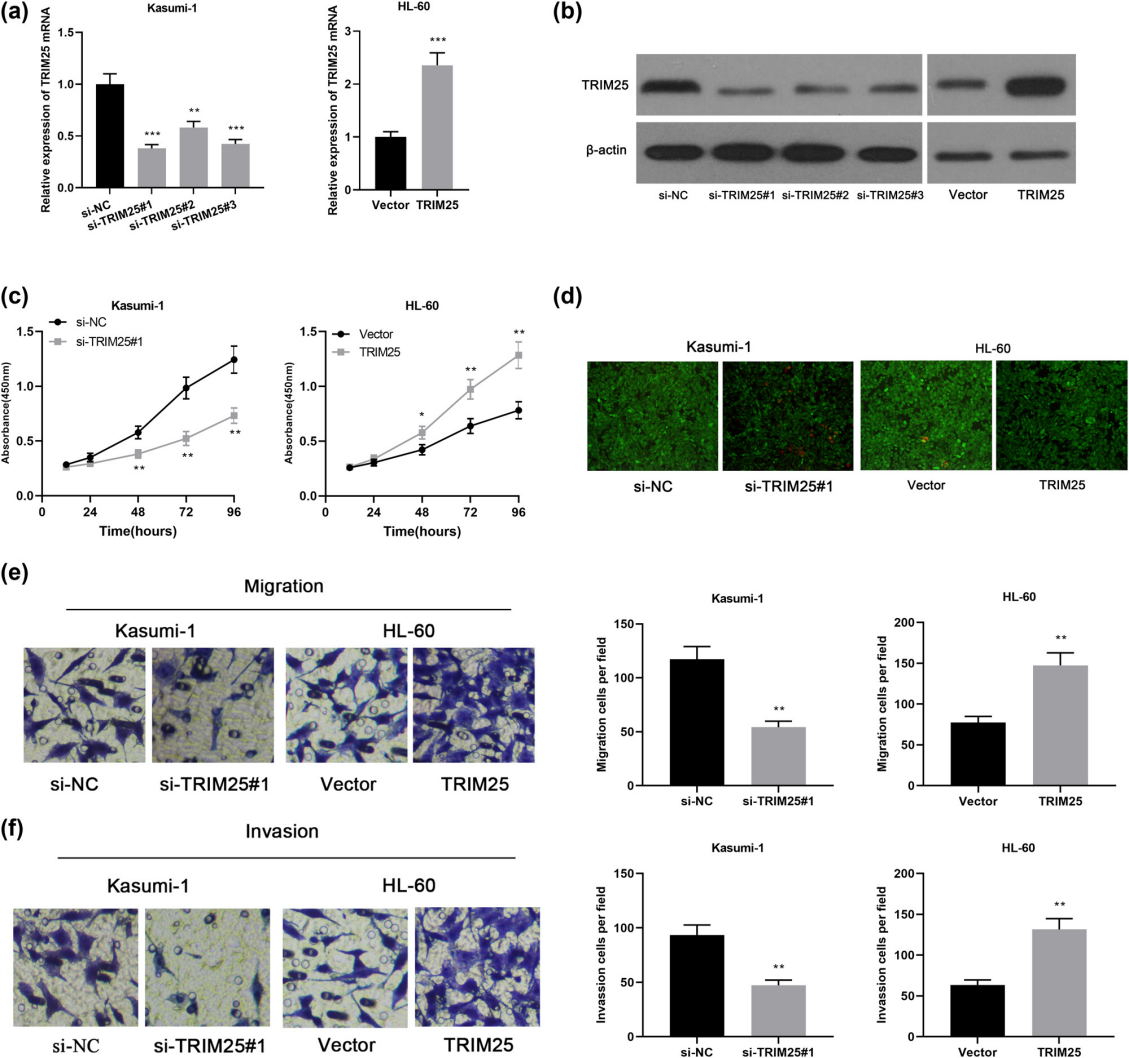
TRIM25 promoted the proliferation, migration, and invasion of AML cells. (a) qPCR to detect TRIM25 mRNA expression in Kasumi-1 and HL-60 cells after knocking down or overexpressing TRIM25. (b) Western blot analysis of TRIM25 protein expression in cells transfected with miR-137 mimics or inhibitor. (c) CCK-8 assay to detect the proliferation of AML cells after knocking down or overexpressing TRIM25. (d) Cell viability was tested using live (calcein staining, green)/dead (PI staining, red) assay. Scale bar = 100 µm. (e and f). Transwell test to detect the migration and invasion of AML cells after knocking down or overexpressing TRIM25. *P < 0.05, **P < 0.01, ***P < 0.001.

To study the impact of TRIM25 on the malignant progression of AML, we transfected TRIM25 siRNAs in Kasumi-1 cells with the highest TRIM25 expression to construct a TRIM25 knockdown cell model, and transfect TRIM25 in HL-60 cells with the lowest TRIM25 expression. The expression plasmid was used to build a cell model of TRIM25 overexpression, and the transfection effect of the cells was assayed with qPCR. TRIM25 expression in Kasumi-1 cells transfected with TRIM25 siRNAs was reduced, whereas TRIM25 expression was observably upregulated in HL-60 cells transfected with TRIM25 overexpression plasmids (Figure 2a). Western blot analysis of TRIM25 protein showed that siRNA silencing markedly reduced TRIM25 protein expression, and TRIM25 expression construct significantly increased TRIM25 protein (Figure 2b). We further used the CCK-8 experiment, Calcein-AM/PI double staining, and the Transwell experiment to detect cell proliferation, migration, and invasion. Consequently, knocking down TRIM25 significantly inhibited the invasion, migration, and proliferation of Kasumi-1 cells compared to the control group, whereas overexpression of TRIM25 significantly promoted the invasion, migration, and proliferation of HL-60 cells (Figure 2c–f). The above certificated that TRIM25 exerts a carcinogenic function in the progress of AML via facilitating the AML cell’s proliferation, invasion, and migration.

### 3.3 TRIM25 was a downstream target of miR-137

**Figure 3:**
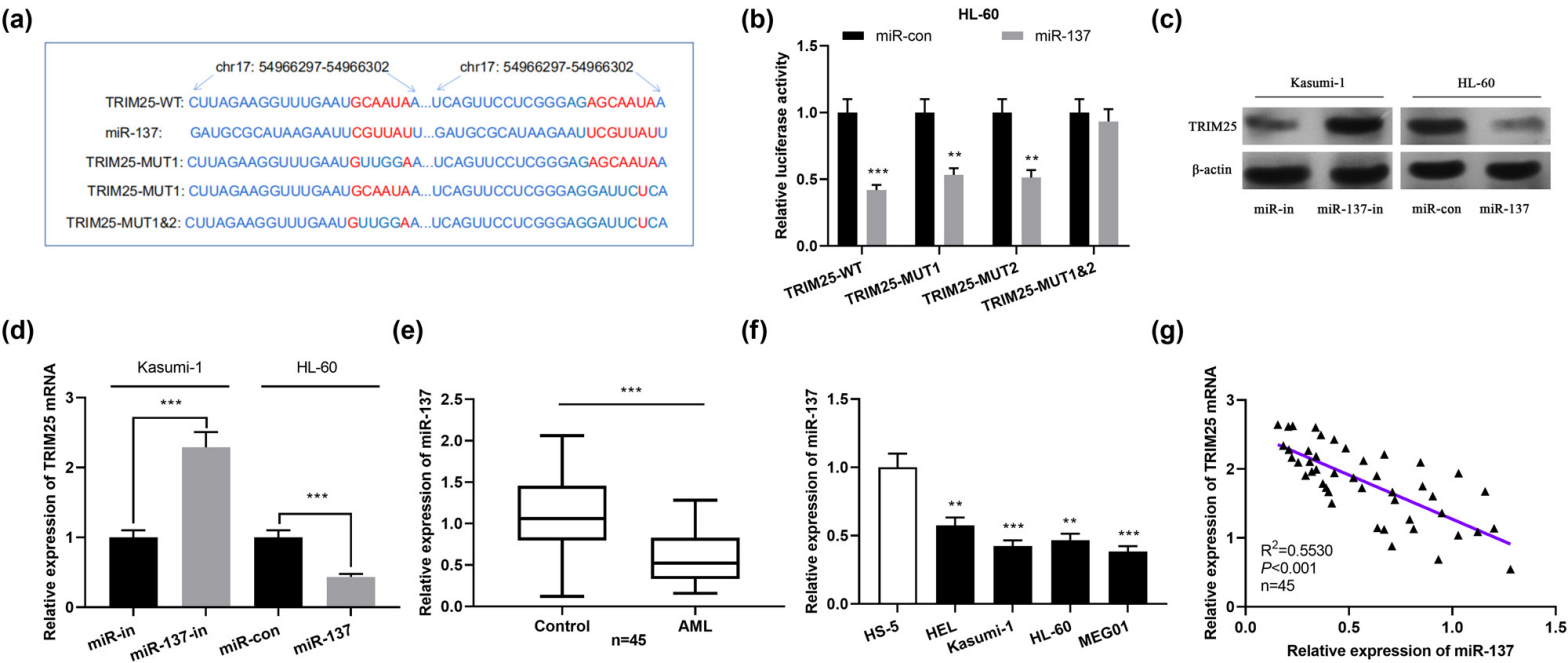
TRIM25 was the target of miR-137. (a) Bioinformatics analysis predicted the binding sequence of miR-137 and TRIM25. (b) Dual-luciferase reporter experiment to analyze the binding relationship between miR-137 and TRIM25. (c and d) Western blot and qPCR to detect the effect of knockdown or overexpression of miR-137 on TRIM25 protein and mRNA expression. (e and f) qPCR to detect miR-137 expression in AML samples and cells. (g) qPCR to evaluate the correlation between miR-137 and TRIM25 mRNA expression in AML samples. **P < 0.01, ***P < 0.001.

To further clarify the potential causative role of TRIM25 in the progress of AML, bioinformatics analysis was carried out to predict the miRNA targets of TRIM25 using starBase (http://starbase.sysu.edu.cn/) and miRanda (http://www.microrna.org/). Among these miRNAs, miR-137, which is a tumor-suppressive miRNA in various cancers, attracted our interest. We verified the presence of a base complementary binding site of miR-137 with TRIM25 (Figure 3a). We further adopted a dual-luciferase reporter experiment for assaying the targeting association of TRIM25 with miR-137. As a result, overexpression of miR-137 visibly reduced wild-type TRIM25 luciferase activity but harbored no critical influence on the luciferase activity of mutant TRIM25 (Figure 3b). Western blot and qPCR exhibited that miR-137 knockdown increased TRIM25 protein and mRNA expression, whereas overexpression of miR-137 inhibited TRIM25 mRNA and protein expression (Figure 3c and d). Besides, qPCR was used for assaying miR-137 expression in AML cells and blood samples. The results revealed significantly downregulated miR-137 in AML cell lines and blood samples (Figure 3e and f). TRIM25 mRNA level in AML blood samples harbored a negative association with miR-137 (Figure 3g). These consequences implied that TRIM25 is the target of miR-137 in AML cells, and the negative feedback of miR-137 regulates its expression.

### 3.4 miR-137 restrained AML cell’s invasion, migration, and proliferation by modulating TRIM25

**Figure 4:**
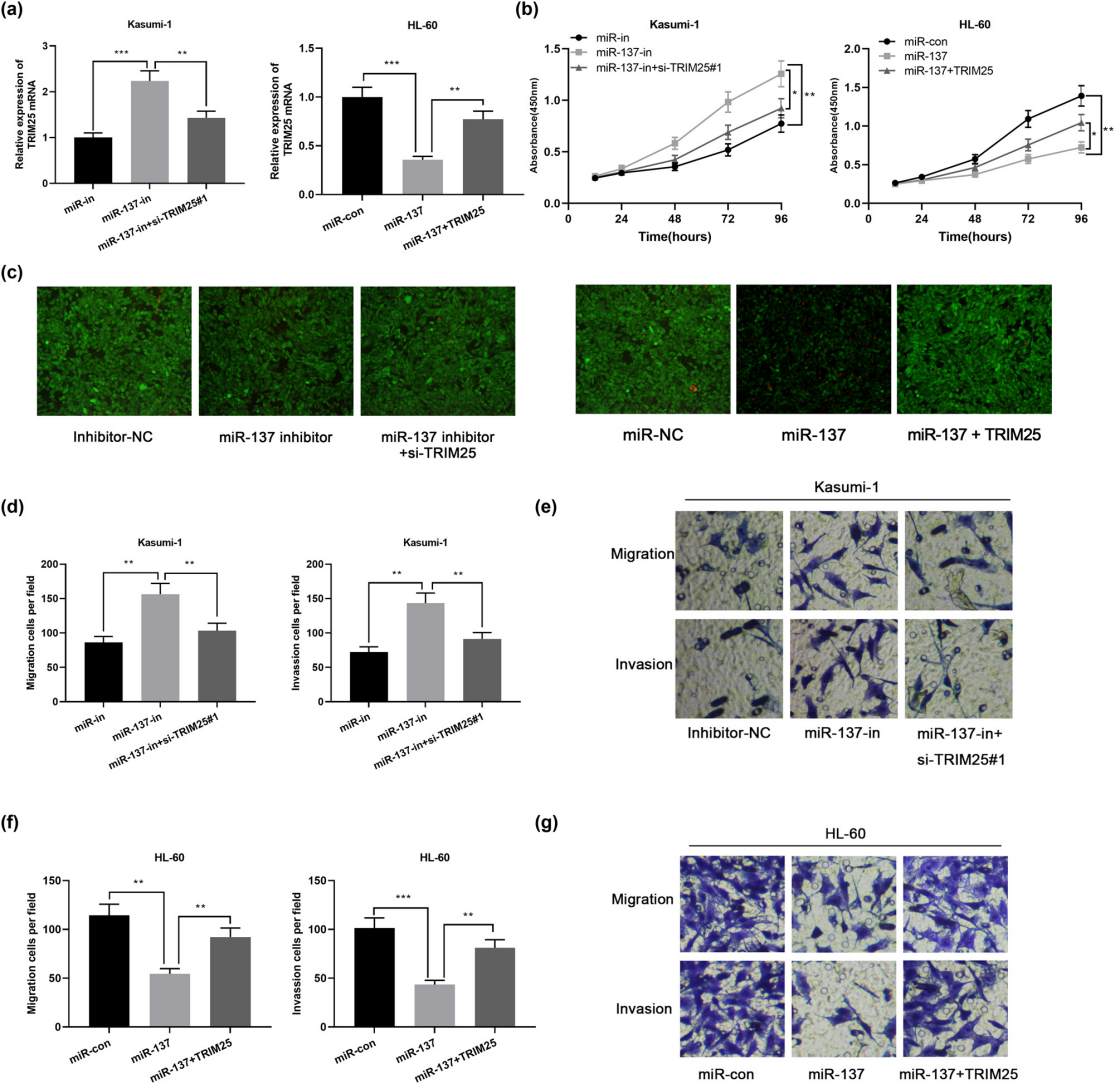
miR-137 inhibited AML cell’s proliferation and migration by targeting TRIM25. (a) qPCR to detect TRIM25 mRNA expression in Kasumi-1 cells transfected with miR-137 inhibitor or co-transfected with si-TRIM25#1 and HL-60 cells transfected with miR-137 mimics or co-transfected with TRIM25. (b) CCK-8 assay was performed to detect cell proliferation. (c) Cell viability was visualized by staining the cytoplasm with a live cell indicator, Calcein-AM (green). Scale bar = 100 µm. (d–g) Transwell assay to detect cell migration and invasion. *P < 0.05, **P < 0.01, ***P < 0.001.

To clarify the mechanism of action of miR-137 in AML, we transfected Kasumi-1 cells with miR-137 inhibitors or co-transfected with si-TRIM25#1, and transfected miR-137 mimics in HL-60 cells or transfect miR-137 + TRIM25, and used qPCR to detect the efficiency of cell transfection (Figure 4a). Further, AML cell’s proliferation, invasion, and migration were assayed adopting the CCK-8 assay, Calcein-AM/PI double staining, and Transwell assay. Consequently, in contrast to the control group, miR-137 knockdown facilitated AML cell’s invasion, migration, and proliferation, whereas the downregulation of TRIM25 attenuated this promotion effect (Figure 4b–f). On the contrary, overexpressed miR-137 notably suppressed HL-60 cells invasion, migration, and proliferation, and this inhibitory effect was remarkably reversed by overexpressed TRIM25 (Figure 4b–g). These outcomes implicated that miR-137 suppresses AML cell’s migration, invasion, and proliferation via modulating TRIM25.

## 4 Discussion

The low early diagnosis rate, the fast progress, and poor curative effect result in an unfavorable prognosis of patients with AML [18,19]. Therefore, exploring the biological mechanism of how AML develops is imperative. In this work, we evidenced that TRIM25 is upregulated in AML. The miR-137/TRIM26 axis regulated AML cell’s invasion, migration, and proliferation, promising new latent treatment strategies for patients with AML.

TRIM25, which belongs to the TRIM protein family, has RBCC multi-domains, encompassing RING, coiled-coil, and B-box. As E3 ligase, it can be combined with substrate protein to make it ubiquitinated/ubiquitin-like degradation or modification, thereby regulating the biological function process [5,6,7]. Many studies have shown that the TRIM family has antiviral effects and actively participates in the modulation of cell apoptosis, differentiation, and proliferation [7]. Furthermore, increasing research evidence shows that TRIM family proteins are essential regulators of tumorigenesis and development and play crucial functions in the biological signs of progress comprising tumor cell differentiation, proliferation, apoptosis, invasion, and migration, among which TRIM19, TRIM24, TRIM33, and TRIM2 mainly obtain the oncogene characteristics through chromosomal rearrangement [6], TRIM27 downregulates its tumor-suppressive effect by mediating the atypical ubiquitination of the tumor suppressor gene PTEN [20]. Studies have shown that TRIM25 can be used as a transcription factor to regulate cellular biological processes and exert a carcinogenic function in carcinoma progress. For instance, TRIM25 is markedly overexpressed in gastric carcinoma, and TRIM25 knockdown suppresses the migration and invasion of gastric carcinoma cells via the TGF-β signal [21]. TRIM25 contributes to the survival and the growth of hepatoma cells via targeting the Keap1-Nrf2 pathway, and highly expressed TRIM25 predicts the unfavorable prognosis of patients with liver carcinoma [22]. In this study, we confirmed the cancer-promoting role of TRIM25 in AML and found that TRIM25 is overexpressed in AML blood samples and cells.

Further functional experiments confirmed that knocking down TRIM25 suppressed AML cells’ proliferation, invasion, and migration, whereas overexpressed RIM25 facilitates cell invasion, migration, and proliferation. In addition, the high TRIM25 expression was distinctly concerned with the unfavorable prognosis of patients with AML. These findings implicated that TRIM25 could participate in the progression of AML as a carcinogen.

Some studies have found that miRNA is a significant factor in diverse physiological progress encompassing cell differentiation, apoptosis, and proliferation [23]. It is often dysregulated in various human tumors and is closely connected to tumor advancement [24]. The research found a certain level of miRNA in human serum, and the change of miRNA expression profile in serum has a clear correlation with the progress of various tumors [25,26]. Substantial studies have confirmed that miRNA exerts a crucial regulatory function in tumor progress (including AML) and participates in inhibiting or promoting tumors’ malignant progression. For example, miR-34c-5p inhibits exosome shedding via selectively targeting RAB27B, induces AML cell senescence, and promotes the elimination of AML stem cells [27]. miR-34a contributes to AML cell apoptosis and restrains autophagy via targeting HMGB1 [28]. miR-485-5p suppresses the AML cell proliferation via targeting SALL4 [29]. In this work, we evidenced significantly downregulated miR-137 in AML blood samples as well as cells. *In vitro* cell function experiments confirmed that knocking down miR-137 significantly raised AML cell’s invasion, migration, and proliferation abilities. The overexpressed miR-137 inhibited the AML cell’s invasion, migration, and proliferation. These detections implicated the tumor inhibition function of miR-137 in AML.

Studies revealed that miRNA is capable of playing a critical part in cancer progression by regulating TRIM25. For example, miR-365 restrains non-small cell lung carcinoma’s progress by suppressing TRIM25 expression [30]. Overexpressed miR-3614-3p inhibits the growth of breast carcinoma cells via downregulating TRIM25 [31]. It is worth mentioning that this study found the existence of a binding site of TRIM25 with miR-137 using bioinformatics analysis. Given previous research, we speculate that miR-137 likely plays a part as an upstream molecule of TRIM25 in AML. In the dual-luciferase reporter experiment, we showed that miR-137 is capable of directly binding to TRIM25, and the two expressions harbored a negative correlation in AML blood samples. In addition, knocking down TRIM25 attenuated the promotion of AML cell’s invasion, migration, and proliferation because of silencing miR-137. On the contrary, overexpression of TRIM25 significantly reversed the suppressive impact of overexpression of miR-137 on AML cell’s migration, invasion, and proliferation, indicating that miR-137 can target TRIM25 to regulate AML cell’s proliferation, invasion, and migration.

To conclude, our study evidenced highly expressed TRIM25 in AML blood samples and cells, which is concerned with the unfavorable prognosis of patients with AML. Besides, we confirmed that the miR-137/TRIM25 axis takes part in modulating AML’s cell invasion, migration, and proliferation. This study reveals new potential molecular mechanisms in the progress of AML and provides an original rationale for clinically diagnosing and treating AML.
